# “Botched”: A Case Report of Silicone Embolism Syndrome After Penile and Scrotal Injection

**DOI:** 10.5811/cpcem.2020.9.48838

**Published:** 2020-10-20

**Authors:** Anantha Singarajah, Albert Wang, Julie Sayegh, Gary M. Vilke, Faith C. Quenzer

**Affiliations:** * Lincoln Memorial University – DeBusk College of Osteopathic Medicine, Harrogate, Tennessee; † Desert Regional Medical Center, Department of Emergency Medicine, Palm Springs, California; ‡ Los Alamitos Medical Center, Department of Emergency Medicine, Los Alamitos, California; § University of California, San Diego, Department of Emergency Medicine, San Diego, California

**Keywords:** Silicone embolism syndrome, illicit silicone injections, injected liquid silicone, acute respiratory failure, acute respiratory distress syndrome

## Abstract

**Introduction:**

Silicone has been commonly used for both major and minor plastic and reconstructive surgery for decades. Due to the high costs associated with minor cosmetic procedures and plastic surgery, the unauthorized use of silicone injections by laypersons has become increasingly common. Improper or illegal subcutaneous injectable silicone has caused significant pulmonary complications and neurological complications, which can range from mild chest pain, hypoxia, and respiratory failure to coma and altered mental status.

**Case Report:**

We present a patient who had a rare complication of respiratory failure secondary to silicone embolism syndrome (SES). SES is a rare, potentially deadly complication and has been associated with subcutaneous silicone injections. The diagnosis of SES can be challenging and requires a thorough patient history indicating recent cosmetic procedures.

**Conclusion:**

This case describes the first case of SES of a male patient who presented to a community emergency department complaining of dyspnea after an episode of self-administered injectable silicone into his penis and scrotum and who developed SES-induced respiratory failure.

## INTRODUCTION

Silicone liquid is a mostly inert, liquid polymer that is commonly used for plastic and reconstructive surgery due to its resistance to heat and aging, and low immune response. Subcutaneous injections of silicone are frequently administered mainly in the breast and buttock area for cosmetic augmentations.^1^–^2^ However, there are reports of injected liquid silicone that resulted in the migration of the silicone, resulting in serious respiratory complications such as silicone embolism syndrome (SES). Symptoms of SES may include dyspnea, fever, cough, hemoptysis, chest pain, hypoxia, alveolar hemorrhage, and altered level of consciousness.^3^ Patients presenting with these clinical features have often been diagnosed with pulmonary embolism, acute respiratory distress syndrome (ARDS), alveolar hemorrhage, and pneumonitis based on radiographic imaging. To our knowledge this is the first case of a male patient who self-injected silicone directly into his penis and scrotum who presented to the emergency department (ED) with acute dyspnea and respiratory failure due to SES.

## CASE REPORT

A 59-year-old, otherwise healthy male presented to the ED with a complaint of acute onset, non-exertional shortness of breath that began six hours prior to arrival. The patient reported that he had injected approximately 80 milliliters (mL) of liquid silicone into his penis and scrotum for purposes of penile and scrotal enlargement approximately one hour prior to the onset of his symptoms. He noted that he rapidly began to experience fatigue and shortness of breath, as well as lightheadedness, cough, and exertional dyspnea. He denied any other symptoms. The patient also reported that he had been self-injecting 80–100 milliliters (mL) of silicone with lidocaine into his genitals regularly since June 2008.

The patient explained that he was part of a group of “brothers” who engaged in the same subcutaneous silicone injection practices. He stated that in the same group there were others who had been previously diagnosed with silicone pulmonary embolism and some of whom had died. Thus, when he started developing symptoms, he contacted his “group mentor” who encouraged him to come to the ED for further evaluation. He denied injecting silicone intravascularly and reported that he had aspirated the needle prior to the injection to make sure it was not in a blood vessel. The patient denied any penile pain or urinary hesitancy or dysuria. He also admitted to injection of 100 mL of silicone into his nipple areas several years prior but denied any recent injection. He denied smoking or illicit drug use, allergies, or any significant family history. His surgical history was only pertinent for a lumbar fourth and fifth discectomy. He uses 50 milligrams (mg)/mL testosterone intramuscular injections every two weeks and emtricitabine/tenofovir as needed.

On physical exam, his initial vital signs showed a temperature of 36.9 degrees Celsius, heart rate of 102 beats per minute, respiratory rate of 22 breaths per minute, blood pressure of 125/73 millimeters of mercury (mm Hg), and an oxygen saturation (SpO_2_) of 82% on room air. The patient appeared to be anxious and in mild respiratory distress. He was diaphoretic, with increased work of breathing with shallow, labored breaths. His lung sounds were notable for diffuse coarse rales and rhonchi throughout the upper and lower lobes bilaterally. His genitourinary exam revealed a significantly enlarged scrotum, approximately 20 centimeters (cm) in diameter, and circumferentially enlarged penile shaft to approximately 6 cm in diameter. The area was firm, without fluctuance, tenderness, erythema or warmth. The penile head appeared normal in size.

The patient’s pertinent laboratory data revealed white blood cell count of 14.1 × 10^9^/ liter (L) (normal 4.0–10.0 × 10^9^/L), hemoglobin of 14.3 grams (g)/dL (normal 13.0–7.0 g/dL), and platelets of 215 × 10^9^/L (normal 150–400 × 10^9^/L). The basic metabolic panel was unremarkable. Arterial blood gas revealed a pH of 7.46 (normal 7.35–7.45), partial pressure of carbon dioxide of 33 mm Hg (normal 35–45 mm Hg), partial pressure of oxygen of 71 mm Hg (normal 75–100 mm Hg), bicarbonate of 23.5 millimoles (mmol)/L (normal 18–22 mmol/L), and a base excess of 0.4 milliequivalents (mEq)/L (normal [−3] – [+3] mEq/L). Prothrombin time, international normalized ratio, and partial thromboplastin time were 12.2, 1.1, and 25.3 seconds, respectively. The urine drug screen was negative for drugs of abuse. The patient’s electrocardiogram revealed a normal sinus rhythm without ischemic changes.

**Image 1:**
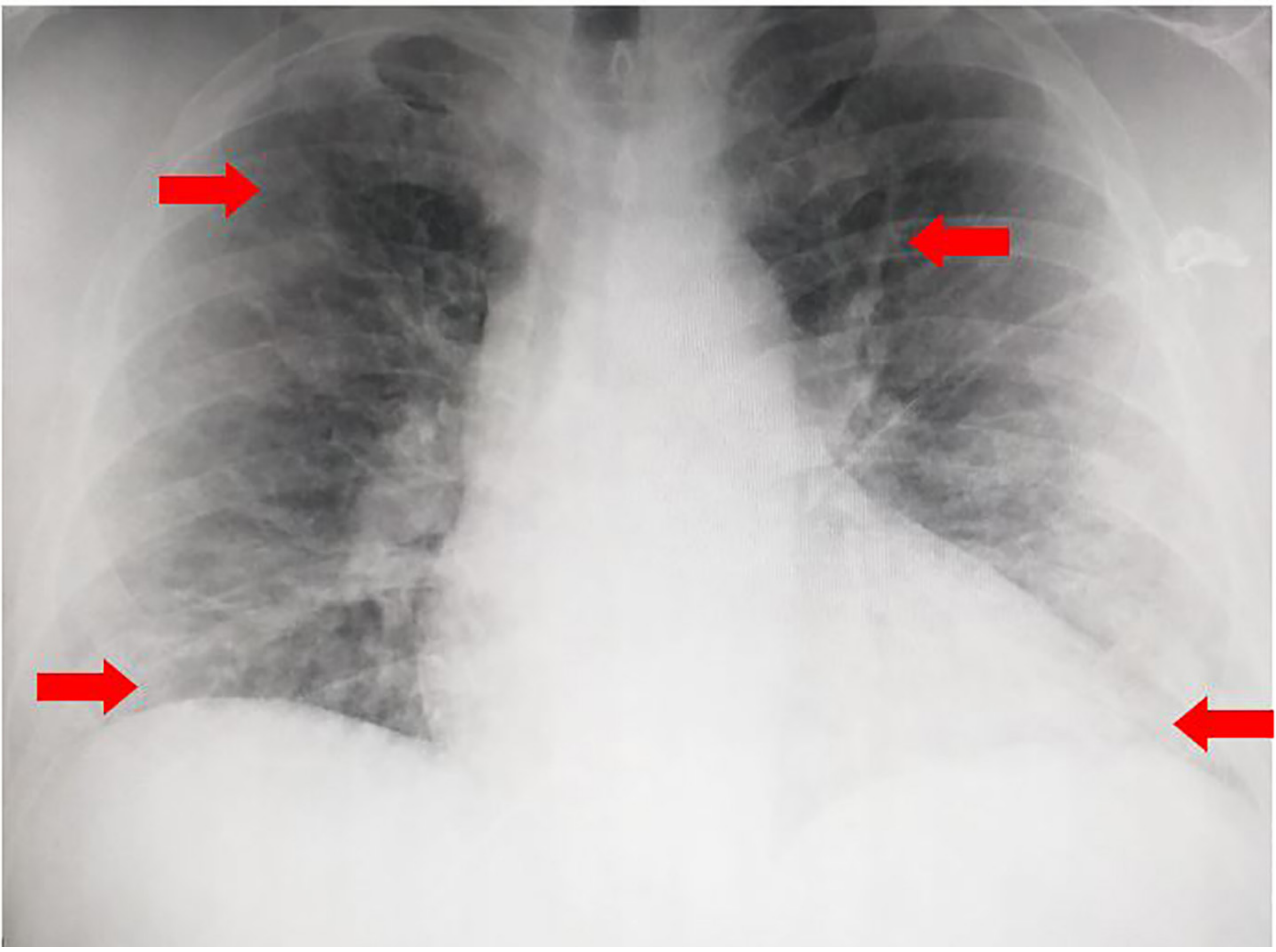
Initial chest radiograph demonstrating mild pulmonary vascular congestion with mild, hazy bilateral airspace disease, likely secondary to alveolar edema or aspiration (arrows).

**Image 2:**
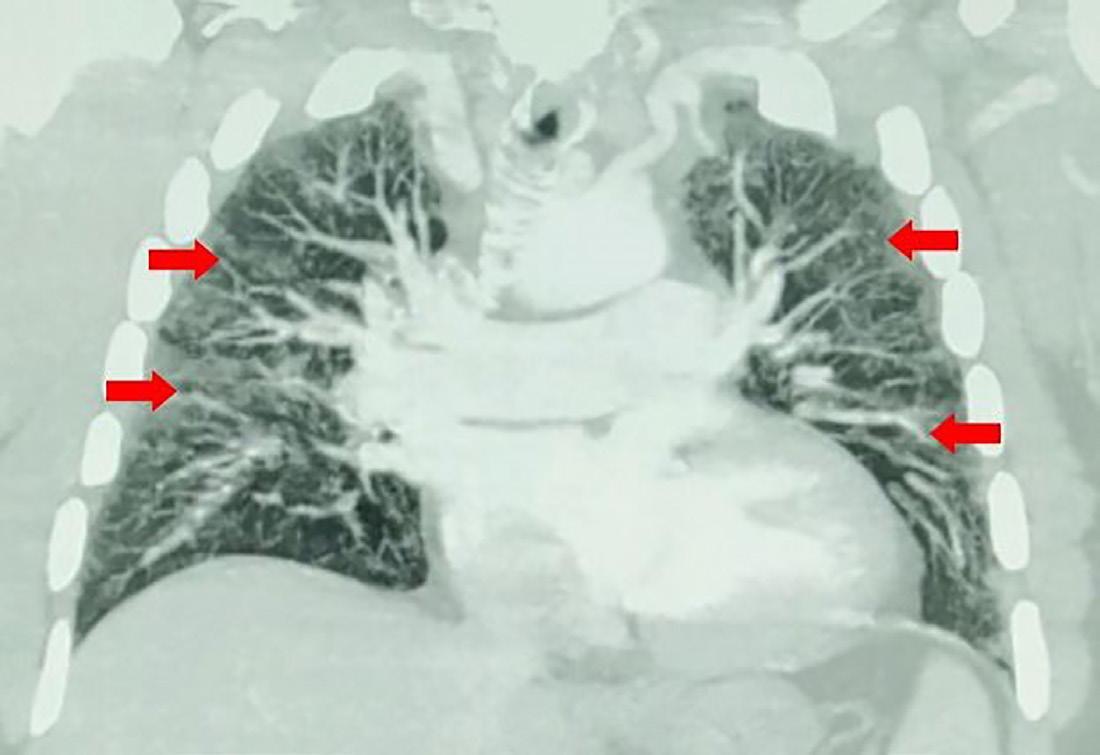
Computed tomography angiography of the chest with intravenous contrast in the coronal view demonstrates no pulmonary embolism. However, there are moderate, scattered, diffuse pulmonary ground-glass and interstitial lung markings. The red arrows indicate mild subcutaneous stranding and edema of the pleura.

**Image 3:**
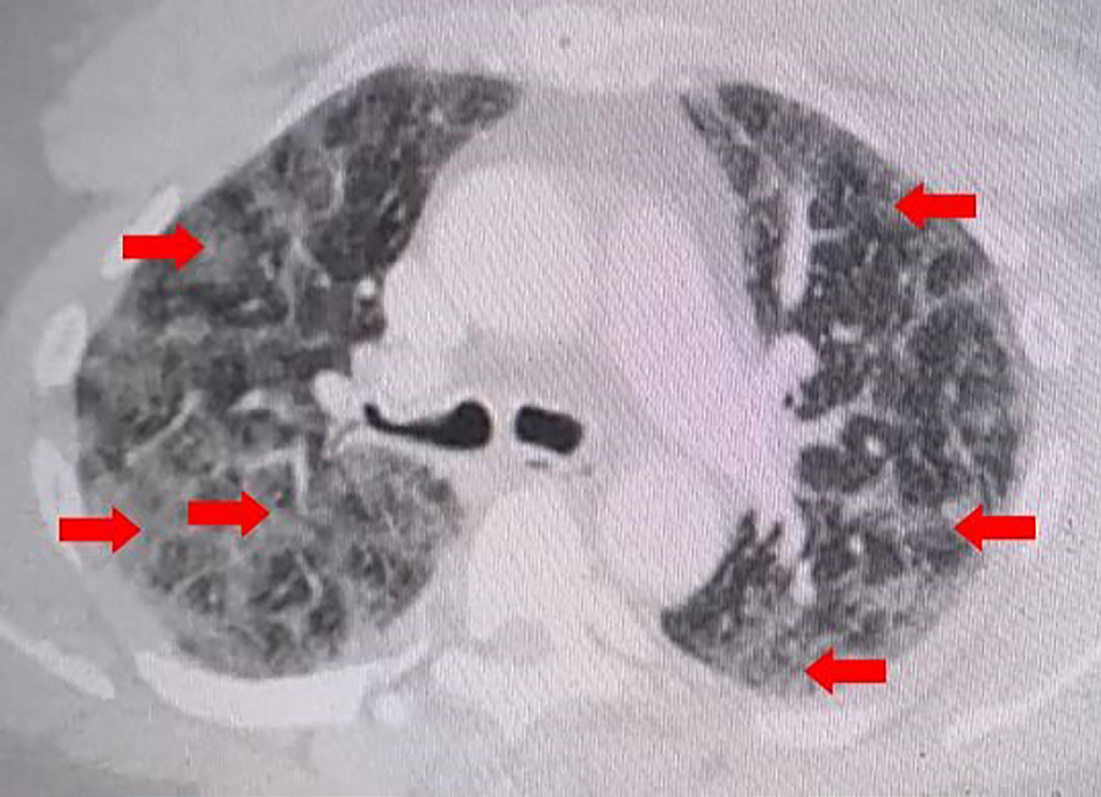
Computed tomography angiography of the chest with intravenous contrast in the transverse view demonstrates moderate, scattered, diffuse pulmonary ground-glass and interstitial lung markings without pulmonary embolism as indicated by the red arrows. There is also mild subcutaneous stranding and edema of the anterior chest wall.

The patient was placed on supplemental oxygen via nasal cannula, and his SpO_2_ improved from 82% on presentation to 100%. A chest radiograph was performed that revealed bilateral alveolar infiltrates (Image 1). Point-of-care cardiac ultrasound was performed, which revealed an enlarged right ventricle with minimal mid-chamber collapsibility, indicating right ventricular heart strain. A computed tomography with angiogram (CTA) of the chest was obtained and demonstrated moderate, scattered, diffuse pulmonary ground-glass and interstitial lung markings consistent with alveolar edema vs bronchopneumonia or ARDS (Images 2 and 3). The CTA chest was negative for pulmonary embolism. There was also mild subcutaneous stranding and edema of the anterior chest wall.

CPC-EM Capsule

What do we already know about this clinical entity?

*Silicone embolism syndrome (SES) is a rare but potentially lethal complication that can occur following cosmetic silicone injections*.

What makes this presentation of disease reportable?

*This is the first reported case of SES in a male patient who injected silicone into his penis and scrotum and ultimately succumbed to respiratory failure*.

What is the major learning point?

*SES should be considered in the differential diagnosis in a patient presenting with dyspnea and hypoxia after cosmetic injections of silicone*.

How might this improve emergency medicine practice?

*An awareness and understanding of SES following silicone injections may potentially improve morbidity and mortality with early detection*.

The patient was admitted to the inpatient telemetry unit due to concern for possible silicone pneumonitis and was started on intravenous methylprednisolone 60 mg every six hours to treat pulmonary inflammation. A scrotal ultrasound (Image 3) was non-diagnostic due to the heavy acoustic shadowing created by the silicone within the scrotum. Urology was consulted regarding the patient’s scrotal swelling and determined that there was no underlying penile or scrotal infection from the silicone injections. The patient was instructed to halt all silicone penile and scrotal injections. Two days into his admission, he became increasingly dyspneic and hypoxic. His oxygen requirements were increased, and he was subsequently intubated and transferred to the intensive care unit. He was diagnosed with ARDS secondary to SES. He continued to be ventilator dependent, had percutaneous tracheostomy and gastrostomy tube placement, and was subsequently transferred to a long-term care facility.

## DISCUSSION

The first report of SES was found in a breast augmentation surgery in 1978 as described by Celli and colleagues.^4^ Since then, cases of complications due to illicit cosmetic silicone injection administration has continued to grow due to increased demand and lack of affordability of medically administered injections.^2^,^5^–^7^ In the United States, silicone injections are frequently administered most commonly in the breast and buttock area in women and transgender individuals.^2^,^8^ It is estimated that that there is a 1–2% incidence rate of silicone-injection complications.^9^ These complications typically present within 48 hours after injection but can occur months later.^5^,^10^

The most common reported presenting symptoms of SES are the following: hypoxia (92%); dyspnea (88%); fever (70%); alveolar hemorrhage (64%); and cough (52%).^2^,^5^ Additionally, neurological manifestations of SES such as altered levels of consciousness and coma have been reported and are poor prognostic indicators.^5^–^6^,^11^ The exact pathophysiology linking injectable silicone and respiratory symptoms is still fairly unclear. It is generally considered that SES is due to the injected silicone either going directly into the bloodstream or migrating into the bloodstream, leading to an embolic event.^12^–^14^ There is also evidence demonstrating that silicone administration may cause a widespread inflammatory reaction, possibly secondary to the formation of antibodies to silicone.^10^,^15^ Additionally, injection of large volumes of silicone directly into the body tissues, as occurred in our patient, can result in local tissue damage.^5^,^14^

The diagnosis of SES can often be missed in the ED due to a wide differential diagnosis seen on plain films and an incomplete patient history.^2^ The presence of hypoxia and dyspnea often prompts CTA of the chest to rule out pulmonary embolism; thus, SES is most often diagnosed with this modality. Often, SES on CTA chest demonstrates peripherally distributed, ground-glass opacities associated with interlobular septal thickening, similar to what can be observed in some eosinophilic lung diseases and fat embolism syndrome.^2^,^5^,^10^ The clinical findings linked in patients with SES are similar to those found in patients with fat embolisms and alveolar hemorrhage.^2^,^6^,^8^,^15^ Thus, anticoagulants are not indicated for SES as they may worsen alveolar hemorrhage and contribute to a decline in respiratory function.^15^

Treatment is largely supportive starting with the use of supplemental oxygen, while mechanical ventilation is reserved for severe hypoxia secondary to SES.^6^,^10^ There is some research to support the early use of corticosteroids to decrease the severity of SES.^8^,^10^

## CONCLUSION

Widespread use of cosmetic silicone injections can be a cause of silicone embolism syndrome. Most commonly these injections are found in the breast and buttock areas. Due to increased demand and lack of affordability of augmentation procedures, there are reports of increased illicit silicone injections with severe complications. Ours is the first reported case in which a patient injected silicone into his penis and scrotum resulting in acute respiratory failure secondary to SES. The diagnosis of SES should be considered especially in a patient who has a history of recent silicone injection and is exhibiting acute respiratory or neurological symptoms.
